# Dissecting lncRNA-mRNA competitive regulatory network in human islet tissue exosomes of a type 1 diabetes model reveals exosome miRNA markers

**DOI:** 10.3389/fendo.2022.1015800

**Published:** 2022-11-09

**Authors:** Tian Fang, Gong Xue, Wu Jianjun, Long Wei, Zhang Xiaomeng, Yang Fan

**Affiliations:** ^1^ Department of Cardiology, The First Affiliated Hospital of Harbin Medical University, Harbin, China; ^2^ Harbin Center for Disease Control and Prevention, Harbin Municipal Health Commission, Harbin, China; ^3^ Department of Cardiology, The Second Affiliated Hospital of Harbin Medical University, Harbin, China; ^4^ College of Bioinformatics Science and Technology, Harbin Medical University, Harbin, China; ^5^ Key Laboratory of Myocardial Ischemia, Ministry of Education, Harbin, China

**Keywords:** type 1 diabetes, exosomes miRNA, network, tissue exosomes, GEO database

## Abstract

**Background:**

Emerging evidence shows that exosomes play a crucial role in the occurrence and development of diabetes and its complications. The molecules in exosomes can be regarded as important markers for the diagnosis of diseases. However, it is presently unclear the pathological association mechanism between exosomes and diabetes.

**Results:**

In this study, transcriptome data and lncRNA regulatory association data of human pancreatic islet-derived exosome were integrated to construct the ceRNA network. Network analysis revealed that lncRNA with differential expression were primarily involved in islet insulin secretion signaling pathways, including Hippo, TGF-beta, Wnt, FOXO, Neurotrophin and ErbB signaling pathway. Further, combined with miRNA mediated competitive regulation and differential expression analysis results, potential markers of diabetes were revealed and validated in independent datasets. Finally, we analyzed the mechanisms of diabetes based on the competitive regulatory association and function of lncRNA.

**Conclusion:**

Our results suggest that lncRNA such as lncRNA PVT1, LINC00960 and hsa-miR-107 might be involved in inflammation response in T1DM, and the former lncRNA chose in the present study may serve as novel biomarkers and potential targets for the diagnosis and treatment of T1DM.

## Introduction

Destruction of β cells is key factor leading to T1DM, which widely affects children and young adults. Besides the burden of T2DM to society, T1DM also poses heavy economic burden on patients and their families. Diabetes is estimated to affect more than 650 million people worldwide by 2040 (1). An urgent need exists for identifying biomarkers to improve the clinical diagnostic process and the therapeutic approach of diabetes (2). Exosomes are generated by nearly all cell types and recent studies have shown that diabetes is associated with alternations in exosomes-mediated communications (3).

With the conclusion of the Human Genome Project and the opening of the post genomic era, long-chain non-coding RNA (lncRNA) has gained lots of attentions as it participates in reprogramming glucose metabolism to diabetes. Suwal et al. demonstrated multiple functions of lncRNA NONRATT021972 in different diabetes-related diseases, including diabetic neuropathy, diabetic cardiac autonomic neuropathy, myocardial ischemia, and hepatic glucokinase dysfunction (4).LncRNA MEG8 is up-regulated in gestational diabetes mellitus (GDM) and contributes to predict kidney injury (5).LncRNA H19 blocks endothelium-mesenchymal transformation in diabetic retinopathy (6).LncRNA KCNQ1OT1 mediates pyrodegeneration in diabetic cardiomyopathy (7).These studies revealed lncRNAs involved in diabetes and were capable of regulating gene expressions *via* diverse mechanisms.

With the continuous deepening research of lncRNAs, its function and mechanism in diabetes and its complications have been further investigated. Competitive regulation of lncRNA is one of the important mechanisms of lncRNA. LncRNA can act as a “sponge” to absorb miRNA, which is called competitive endogenous RNA (ceRNA) (8). In recent years, many studies have focused on the competitive regulation of lncRNA in diabetes. For example, lncRNA DCRF can act as ceRNA to increase PCDH17 expression through sponge absorption of miR-551B-5p, thereby promoting the enhancement of autophagy in diabetic cardiomyopathy (9).LncRNA MALAT1/microRNA let-7F/KLF5 axis regulates podocellular loss in diabetic nephropathy (10). Hu et al. constructed and analyzed abnormal lncRNA-miRNA-mRNA network in adipose tissue of patients with type 2 diabetes mellitus and obesity (11). MiRNAs and lncRNAs not only play important roles in gene expression modules such as gene transcription and post-transcriptional regulation, but also participate in physiological and pathological responses through their interactions in the human body (12). Therefore, an in-depth understanding of the regulatory mechanism based on lncRNA will help us better understand the pathogenesis of diabetes and screen important genes. The aim of our study was to construct lncRNA-related ceRNA networks by integrating lncRNA and mRNA expression data in human pancreatic islet-derived exosome, and compared the regulatory role and functions of lncRNA in control and cytokines treatment exosomes. Among the functional links in ceRNA (lncRNA-miRNA-mRNA axis) regulatory network, a focus on islet-derived exosomes may uncover new regulatory mechanisms of T1DM.

## Materials and methods

### Collection of exosome data from human islet tissue

Gene Expression Omnibus (GEO) Series (GSE) 139932 dataset was retrieved from the National Center for Biotechnology Information (Gene Expression Omnibus, https://www.ncbi.nlm.nih.gov/geo/ ) (13), including expression data of protein-coding genes and non-coding genes from 10 samples in human pancreas-derived islet exosomes. We divided samples into control group (without any cytokines stimulation) and cytokines group (with 50 U/ml IL-1β and 1000 U/ml IFN-γ for 24 hours to mimic the pro-inflammatory T1DM). Exosomes were isolated through ultracentrifugation, combined with different commercial kits. The detailed methods used for exosomes isolation, identification and RNA sequencing have been described on GEO website (https://www.ncbi.nlm.nih.gov/geo/query/acc.cgi?acc=GSE139932). Plasma-derived exosome datasets (GSE 97123, https://www.ncbi.nlm.nih.gov/geo/query/acc.cgi?acc=GSE97123) and saliva exosome datasets (GSE189107, https://www.ncbi.nlm.nih.gov/geo/query/acc.cgi?acc=GSE189107) were also obtained from GEO website accordingly.

### Collection of lncRNA-miRNA and miRNA-mRNA regulatory association data

Global lncRNA-miRNA and miRNA-mRNA interaction were constructed by StarBase V2.0 database (http://StarBase.sysu.edu.cn/), which provides annotations for RNA binding proteins (RBPs) sites, such as lncRNA, mRNA and sncRNA. Meanwhile, StarBase also provides the most comprehensive CLIP-Seq experimentally supported miRNA–lncRNA interaction networks. The effects of 50 U/ml IL-1β and 1000 U/ml IFN-γ stimulation on gene expression were identified in the GSE139932 dataset using the ‘STAR aligner v 2.5.3a’, ‘Gencode v29’ and ‘DESeq2’ packages.

### Construction of ceRNA network

According to the ceRNA theory, lncRNA can bind miRNA by acting as sponges, and then competitively regulate the protein-coding genes targeted by miRNA. In addition, previous studies have also demonstrated that increased expression of lncRNA can enhance expression of the corresponding protein-coding gene. Thus, current researchers generally consider two factors to identify lncRNA-mRNA competitive regulatory relationships: (i) lncRNA and mRNA are targeted by common miRNA (s); (ii) the expressions of lncRNA and mRNA were positively correlated under the studied condition. Based on the above considerations, we identified lncRNA-mRNA competitively regulated relationships as following: first, the candidate lncRNA-mRNA regulatory associations were constructed based on the integration of lncRNA-miRNA and miRNA-mRNA, the significance of shared miRNAs was computed. LncRNA L and mRNA M are considered to be a pair of candidate lncRNA-mRNA regulatory association if the number of miRNAs shared by L and M was more than 3. The ceRNA network was constructed based on the theory that lncRNAs can directly interact by invoking miRNA sponges to regulate mRNA activity. The detailed steps are as follows: for each pair of candidate lncRNA-mRNA, spearman correlation coefficients of lncRNA-mRNA expression were calculated based on samples in different groups. The correlation coefficient is a measure of the degree of correlation between variables, the value is ranging from -1 to 1. A value between 0.8 and 1 is considered to be strongly correlated for variables. In this study, if the correlation value of candidate lncRNA-mRNA in a certain state is greater than or equal to 0.9, the candidate lncRNA-mRNA is considered as a competitive regulatory pair in the corresponding state. In order to ensure the strong correlation between lncRNA and mRNA, we use 0.9 as the correlation cutoff.

### Analysis software

Cytoscape V3.8.0 (Cytoscape Consortium, San Diego, CA, USA, https://github.com/cytoscape/cytoscape/releases/tag/3.8.0 ) software was used to construct and visualize the lncRNA-miRNA-mRNA network. To evaluate the sensitivity and specificity of the identified lncRNA and miRNA biomarkers for distinguishing between control and cytokines treatment groups the total area under the curve (AUC) were calculated using R code.

## Results

### CeRNA network of different states for human islet exosomes

**Figure 1:**
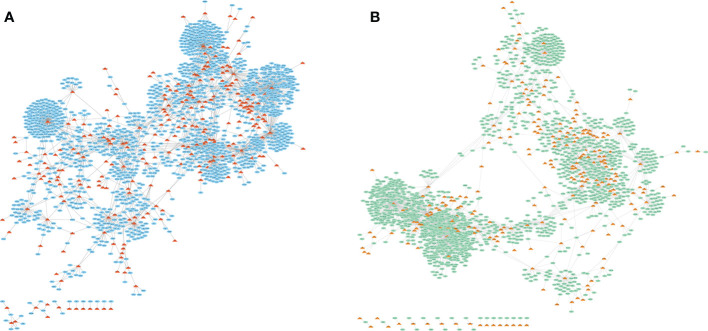
LncRNA-mRNA competitive regulatory network in exosomes for different states treated without **(A)** or with **(B)** 50 U/ml IL-1β and 1000 U/ml IFN-γ stimulation. The triangle represents lncRNAs and the oval shows the total target genes.

LncRNA-miRNA-mRNA axis is a powerful regulatory mechanism and investigated in our study, as we further novelty combined the gene expression data exosomes treated with/without cytokine in human. LncRNA-mRNA competitive regulation networks were constructed in control group and cytokines group, respectively (**Figure 1**). In the GSE139932 dataset, there were 2864 lncRNA-mRNA interactions, 249 LncRNAs and 1560 mRNAs in the competitive regulation network of lncRNA-mRNA in control group; the lncRNA-mRNA competition regulation network contained 2529 lncRNA-mRNA interactions with 244 LncRNAs and 1464 mRNAs in cytokines group. LncRNAs with high degree showed on the two networks such as NEAT1, XIST, MALAT1 and PVT1 (**Tables 1**, **2**).

**Table 1:** The top 10 LncRNAs for degree on the network in control group.

LncRNA	Degree
NEAT1	191
KCNQ1OT1	179
XIST	150
HCG18	101
MALAT1	86
TMEM147-AS1	82
HELLPAR	72
TUG1	66
PVT1	49
NUTM2B-AS1	44

**Table 2:** The top 10 LncRNAs for degree on the network in cytokines group.

LncRNA	Degree
KCNQ1OT1 NEAT1 HELLPAR XIST GABPB1-AS1 CTBP1-DT LINC00665 PVT1 MALAT1 MIRLET7BHG	361 261 139 110 87 77 58 55 53 47

### Functional analysis of competitively regulated mRNAs

**Figure 2:**
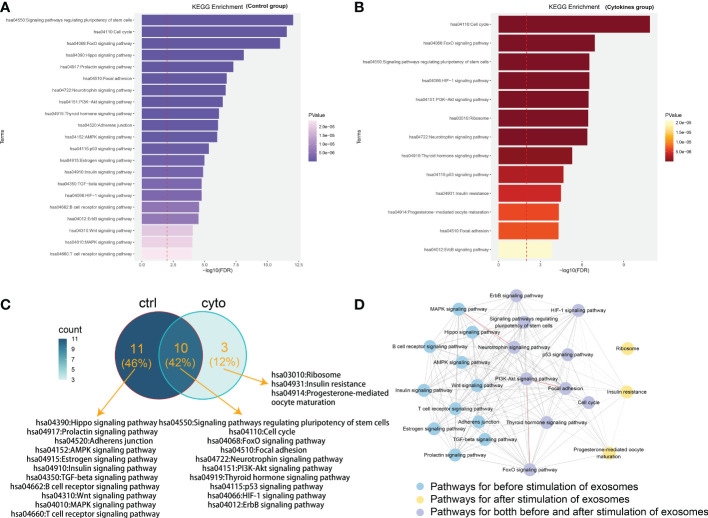
Functional analysis of mRNAs in competitive regulatory networks. **(A)** Gene enrichment function in lncRNA-mRNA network of in control group (without 50 U/ml IL-1β and 1000 U/ml IFN-γ stimulation). **(B)** Gene enrichment function in lncRNA-mRNA network in cytokines group (with 50 U/ml IL-1β and 1000 U/ml IFN-γ stimulation). **(C)** Functional comparison of mRNA enrichment in ceRNA network under exosomes of different states. ctrl: Function enriched for mRNAs in network without cytokines stimulation; cyto: Function enriched for mRNAs in network with cytokines stimulation. **(D)** The pathway crosstalk network. In the crosstalk network, two pathways are connected by an edge if they shared at least one gene. The thickness of the edge is proportional to the number of shared genes between the two connected pathways. Edges colored with red represent the number of shared genes> 20 between the connected two pathways. Pathways with blue, purple and yellow color are specific for different treatment, respectively.

We performed functional enrichment analysis in KEGG database for the mRNA in two lncRNA-mRNA competitive regulation association network. And some state-specific biological functions were enriched in the mRNAs in the state networks with (**Figure 2A**) and without cytokines stimulation (**Figure 2B**).We found that mRNA specific enrichment in the network of exosomes with cytokine stimulation was closely linked to exosomes formation and development of diabetes-related diseases. In particular, we found that these mRNAs were enriched in Hippo signaling pathway, TGF-beta signaling pathway, and Wnt signaling pathway. Mammalian sterile 20-like kinase 1 (Mst1) is an important component of the Hippo pathway, participating in the regulation of organ size, apoptosis and autophagy. As shown in **Figure 2C**, mRNAs were mainly enriched in ten pathways, such as FoxO signaling pathway, HIF-1 signaling pathway, PI3K-Akt signaling pathway and ErbB signaling pathway. Studies have shown that pancreatic cancer cell-derived exosomes induce insulin resistance in C2C12 myotube cells through the PI3K/Akt/FoxO1 pathway (14). Bai et al. demonstrated that exosomes circ_DLGAP4 promotes progression of diabetic nephropathy through competition for miR-143 and further targeted regulation of ERBB3/NF-κB/MMP-2 axis (15). These functions are closely related to the occurrence and process of diabetic cardiomyopathy. Hu et al. found that Mst1-enriched exosomes secreted by cardiac microvascular endothelial cells have pleiotropic effects in cardiomyocytes, including inhibiting autophagy, pro-apoptosis and inhibiting glucose metabolism. TGF-beta pathway has also been confirmed to be involved in a variety of biological processes during exosomes and insulin secretion (16). Zhu et al. demonstrated *in vivo* and *in vitro* that exosomes from macrophages treated with high glucose activate glomerular mesangial cells through the TGF-β1/Smad3 pathway (17).Another important study revealed that mesenchymal stem cell-derived exosomes ameliorated diabetes-induced myocardial injury and fibrosis by inhibiting TGF-β1/Smad2 signaling pathway (18).In addition, Han et al. found that exosomes from autologous skin fibroblasts promote diabetic skin wound healing through the Akt/β-catenin pathway (19). There is functional crosstalk between different biological pathways that closely related with diabetes (20).In order to further understanding the functional roles of lncRNA in the pathological mechanism of type 1 diabetes, we dissected the functional crosstalk between these pathways that enriched by mRNAs in ceRNA networks (**Figure 2D**). In the crosstalk network, two pathways are connected by an edge if they shared at least one gene. We found that PI3K-Akt signaling pathway is enriched by mRNAs on both ceRNA networks. Insulin-activated IRS/PI3K/PKB pathway cascade cross talk could reduce glucose production (20). Previous study has revealed that upregulation of FOXO3 may correlate with the pathogenesis of type 1 diabetes mellitus (21). And PI3K-Akt and FOXO signaling pathways are directly crosstalk with pathways that specifically related in cytokines group (**Figure 2D**). In addition, We did not observe a direct crosstalk between pathways involved before and after stimulation of exosomes. This further suggests that pathways that shared between both states (control and cytokines groups) such as PI3K-Akt and FOXO signaling pathways may play critical roles in contributing to the occurrence of diabetes. Our analysis indicate that the ceRNA network which we constructed is of great significance for revealing the mechanism of lncRNA regulation in exosomes affecting the occurrence and development of diabetes.

### Transcription regulation and ceRNA network

**Figure 3:**
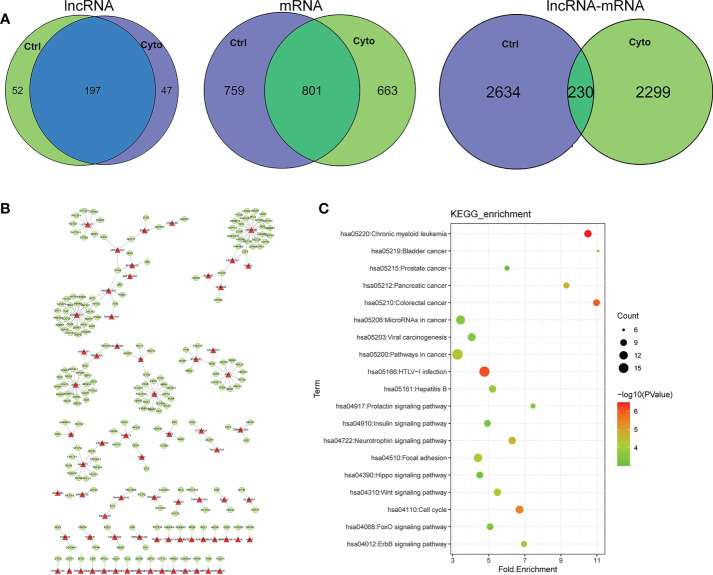
**(A)** Comparison of lncRNA, mRNA and lncRNA-mRNA regulatory relations in different network of exosomes. ctrl: Function enriched for mRNAs in network without cytokines stimulation; cyto: Function enriched for mRNAs in network with cytokines stimulation. **(B)** Competitive regulation of lncRNA-mRNA shared in networks of two states. **(C)** Function of enrichment for mRNAs.

We further analyzed our data the networks in two different states from the perspective of regulatory association of lncRNA, mRNA and lncRNA-mRNA. Venn diagram analysis was performed to obtain differentially expressed genes in the datasets. The lncRNA-mRNA competitively regulated association network constructed in control group and cytokines groups contained 249,244 lncRNAs and 197 lncRNAs, respectively. The lncRNA-mRNA competitive regulatory association networks constructed in exosomes before and after cytokine stimulation contained 1560 mRNAs in control group and 1464 mRNAs in cytokines groups, sharing 801 mRNAs. The lncRNA-mRNA competitive regulation association networks constructed in exosomes in control group and cytokines groups contained 2864,2529 lncRNA-mRNA interactions respectively, and shared 230 lncRNA-mRNA interactions (**Figure 3A**). According to the comparison results, the degree of lncRNA sharing (66.5%) was higher than the degree of mRNA sharing (36.0%) in the network, that is, the two networks were more inclined to share lncRNAs than the mRNAs (**Figure 3**). These results indicate that although some shared lncRNAs play a competitive regulatory role in different states of exosomes, the downstream mRNAs regulated by them may be different in distinct states, that is, the functions involved in regulation are different under different conditions. Meanwhile, we found that the same lncRNA-mRNA shared by the two networks in different treatment was lower (4.4%), which further indicated that LncRNAs in the two different states of exosomes had a great difference in regulation and function. We extracted the interaction association of 230 pairs of ncRNA-mRNAs shared by the two states (**Figure 3B**). We found that lncRNAs involved in the shared association on the two networks included plasmacytoma variant translocation gene (PVT1), NEAT1, XIST and MEG8, etc. Among them, some lncRNAs have been confirmed to be closely associated with the occurrence and development of type 1 diabetes. For example, upregulation of PVT1 contributes to T1DM induced end-stage renal disease (22).LncRNA XIST serves as a diagnostic biomarker of gestational diabetes and regulates trophoblast cells *via* miR-497-5p/FOXO1 axis (23).At the same time, KEGG enrichment analysis was conducted for these lncRNA-mRNA interactions (**Figure 3C**). The node size reflects the degree of enrichment: the smaller the degree value, the smaller the node size is; the color intensity reflects the significance of the enrichment: the darker the pathway node, the more statistically significant it is. We found that these interactions were significantly enriched in exosome related functions, such as insulin signaling pathway, FOXO signaling pathway, Neurotrophin signaling pathway and ErbB signaling pathway (**Figure 3C**).

**Figure 4:**
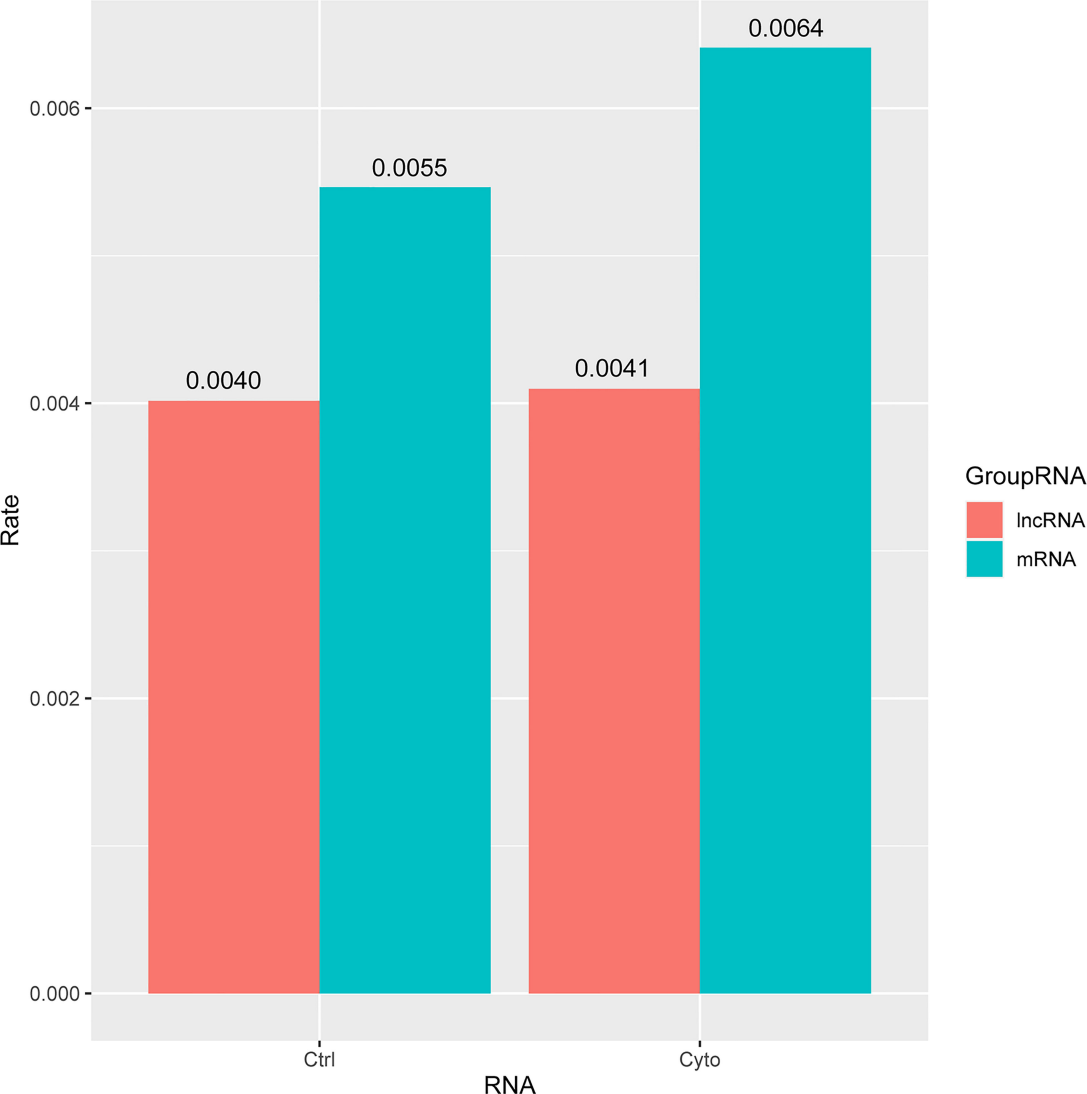
The ratio of differential lncRNAs and mRNAs in the two networks of exosomes. ctrl: Function enriched for mRNAs in network without cytokines stimulation; cyto: Function enriched for mRNAs in network with cytokines stimulation.

Next, we analyzed the differentially expressed lncRNAs and mRNAs in the competitive regulation association network of 2 lncRNA-mRNA in 2 groups. We found that the proportion of differentially expressed lncRNA and mRNA in exosomes was relatively low in control group compared with cytokines group. Meanwhile, the proportion of differentially expressed lncRNA was almost the same (0.4%) with or without cytokines treatment, whereas the proportion of differentially expressed mRNA was higher in cytokines group compared with control group (**Figure 4**).

Our analysis showed that competitive network had a large regulatory difference between control and cytokines group, and the proportion of different lncRNA and mRNA on the network was relatively low. It suggested that the construction of competitive regulatory network could help us to deeply reveal the regulatory molecular mechanism between non-coding RNAs and genes in exosomes. At the same time, it can provide further supplement for the discovery and identification of potential molecular markers in diabetes.

### Differential regulation analysis of shared LncRNAs on the network

**Figure 5:**
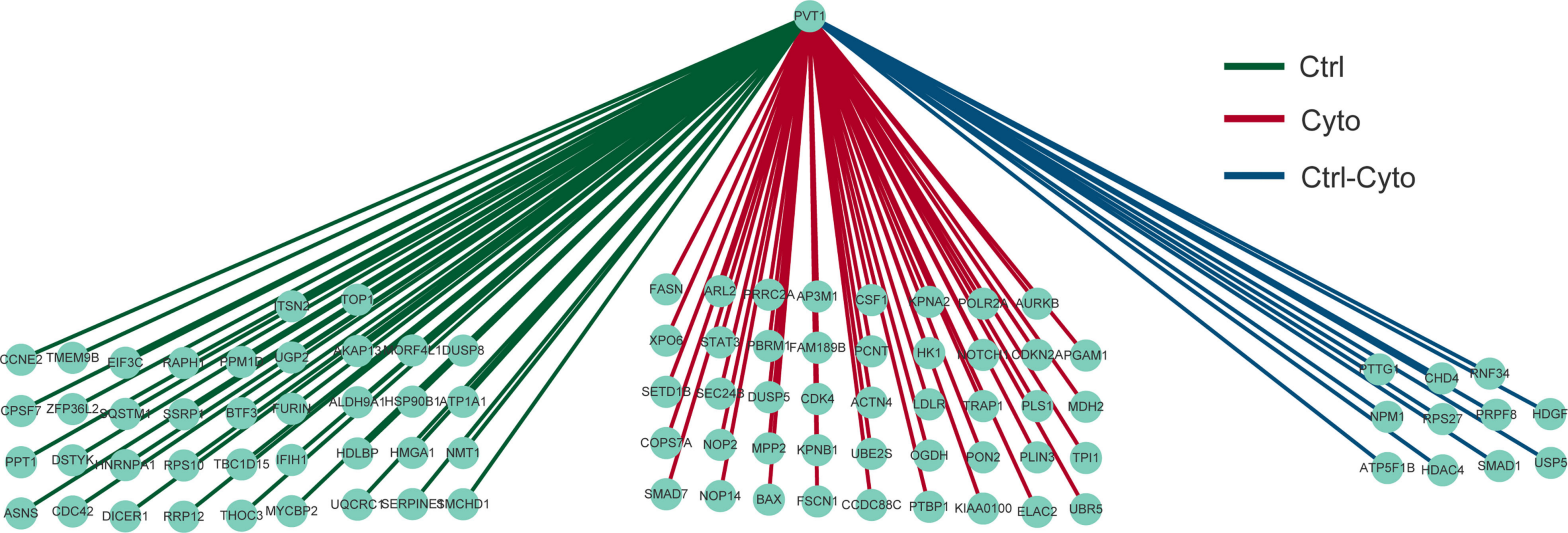
Differential regulation of LncRNA PVT1 with or without cytokines stimulation.

In order to further reveal the differential regulation of lncRNA in type 1 diabetes, we extracted one common and differentially expressed lncRN, as known as lncRNA PVT1, which has been proved to play an important role in exosomes and diabetes. We demonstrated the relationship with mRNA regulated by PVT1 in the two networks among 10 samples as shown in **Figure 5**.The central point is lncRNA PVT1, surrounded by regulatory mRNAs points. We found that PVT1 exists in both networks, indicating that PVT1 may play different functions in physical state, while also contribute to pathological states. In pancreatic cancer, lncRNA PVT1 can promote exosome secretion by YKT6, RAB7 and VAMP3 (24).Wang et al. found that down-regulated PVT1 can lead to gestational diabetes by regulating human trophoblast cells (25).Li et al. found that PVT1 regulates the occurrence and development of diabetic peripheral neuropathy (DPN) by activating the PI3K/AKT pathway in diabetic rats model (26). These studies verified to our construction, which could help researchers to improve the accuracy and reliability of competitive regulation in diabetes.

### MiRNAs involved in competitive regulation in exosomes have potential as diagnostic markers for diabetes

**Figure 6:**
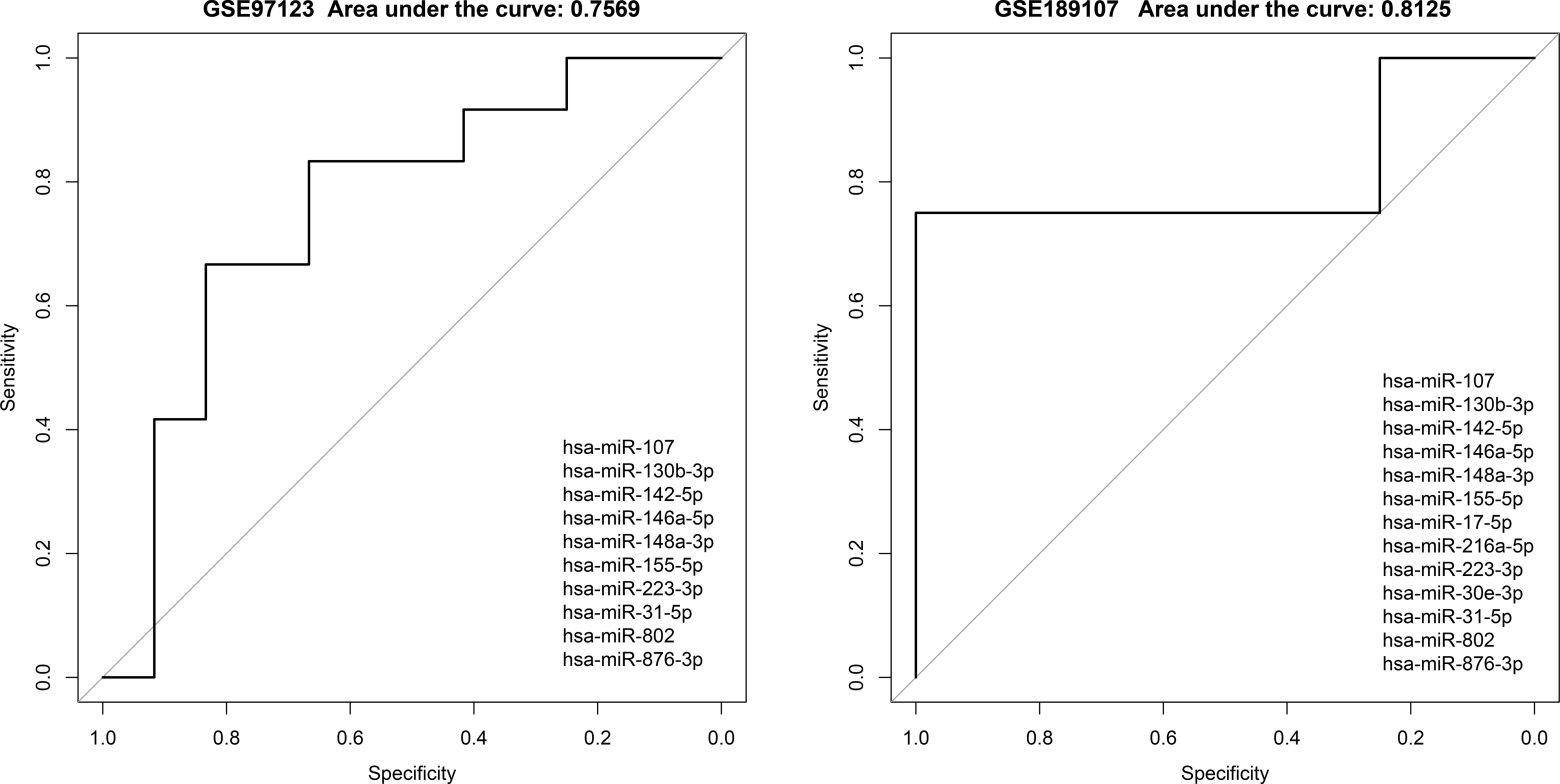
Evaluation of classification effectiveness of competitively regulated miRNAs in exosomes in independent validation sets.

We found evidences of miRNAs extracted from exosomes or blood can be used as markers for diagnosis or even in predicting the occurrence of diseases (27).Therefore, we further extracted miRNAs that potentially mediate lncRNA-mRNA association in competitive regulatory networks, combined with the list of differential miRNAs provided by Krishnan et al. (13). Candidate diabetic miRNA markers were screened, and a total of 19 candidate miRNAs were obtained. Then, we used these miRNAs involved in competitive regulation and differential expression to perform regression analysis in GSE139932 dataset and obtained the risk coefficients of 19 miRNAs (**Table 3**).Finally, we applied the regression coefficient of training to two independent datasets, including plasma-derived exosome datasets (GSE97123) and saliva exosome datasets (GSE189107). There were 10 and 13 miRNAs in the expression spectrum, respectively. ROC curves of independent data sets are shown in **Figure 6**, and AUC are 0.7569 and 0.8125, respectively. This suggests that our model provides more reliable and robust prediction results, which gave the strong evidence in favor of.diabetic mechanisms both in our data and previous studies.

**Table 3:** The coefficient of 19 miRNAs in model.

miRNA	Coefficient
Hsa-miR-107	-284.08
Hsa-miR-130b-3p	250.03
Hsa-miR-142-5p	249.36
Hsa-miR-146a-5p	-116.58
Hsa-miR-148a-3p	624.41
Hsa-miR-155-5p	-181.37
Hsa-miR-17-5p	1947.24
Hsa-miR-216a-5p	-59.90
Hsa-miR-221-5p	-364.84
Hsa-miR-223-3p	93.85
Hsa-miR-30e-3p	54.25
Hsa-miR-31-3p	-192.14
Hsa-miR-31-5p	-103.83
Hsa-miR-4485	-382.17
Hsa-miR-486-3p	-3.99
Hsa-miR-582-3p	595.41
Hsa-miR-758-3p	269.49
Hsa-miR-802	-1044.62
Hsa-miR-876-3p	167.76

### Dissecting lncRNA related regulatory mechanisms in diabetes based on ceRNA network

**Figure 7:**
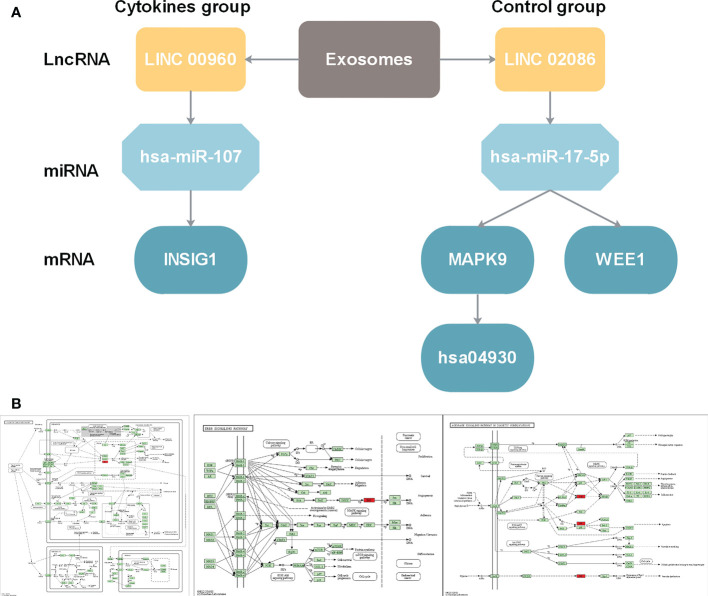
**(A)** LncRNA-miRNA-gene regulation cascades for lncRNA LINC00960 in state after exosome stimulation and for lncRNA LINC02086. **(B)** Function annotation for MAPK9 in KEGG pathways. From left to right: Diabetic cardiomyopathy pathway (hsa05415)(https://www.kegg.jp/kegg-bin/show_pathway?16584585919469/hsa05415.args; ErbB signaling pathway (hsa04012) (https://www.kegg.jp/kegg-bin/show_pathway?16584585919469/hsa04012.args); AGE-RAGE signaling pathway in diabetic complications (hsa04933) (https://www.kegg.jp/kegg-bin/show_pathway?16584585919469/hsa04933.args).

**Figure 8:**
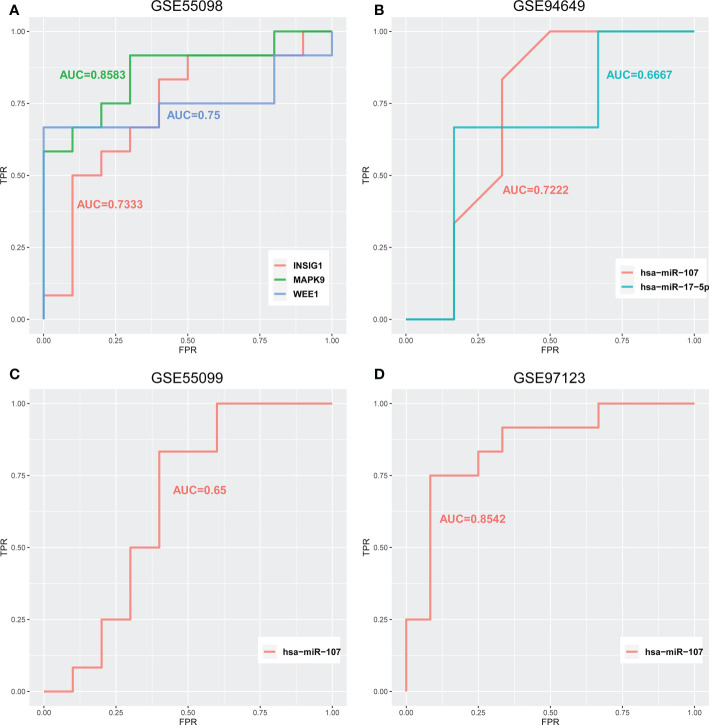
ROC curves for classifying type 1 diabetes samples of **(A)** GSE55098 based on the expression of LINC02086-hsa-miR-17-5p-MAPK9/WEE1 and LINC00960-hsa-miR-107-INSIG. **(B)** GSE94649 based on the expression of LINC02086-hsa-miR-17-5p-MAPK9/WEE1 and LINC00960-hsa-miR-107-INSIG1. **(C)** GSE55099 and **(D)** GSE97123 based on the expression of LINC00960-hsa-miR-107-INSIG1 regulatory axes.

In the above section, we found that differentiated miRNAs that mediate competitive regulation have the potential to serve as diagnostic markers for diabetes. Next, we dissect the related mechanism of diabetes based on the constructed ceRNA network. LINC02086- hsa-miR-17-5p- MAPK9/WEE1 and LINC00960-hsa-miR-107-INSIG1 (**Figure 7A**), in association with the type I diabetes. To further validate the important role of the above two regulatory axes in type 1 diabetes, we explored the gene and miRNA components for classifying samples of type 1 diabetes. We firstly collected gene and miRNA expression profiles of type 1 diabetes from the GEO database (**Figure 7B**), and obtain one gene expression profile (GSE55098) and two miRNA expression profiles (GSE55099 and GSE94649). Furthermore, we also used the above independent blood exosome dataset (GSE97123). We plotted ROC curves for classifying type 1 diabetes sample based on the expressions of each miRNA/gene in the two regulatory axes. As shown in **Figure 8**, AUC values for genes MAPK9,WEE1 and INSIG1 are ranged from 0.7333to 0.8583 (**Figure 8A**), and AUC values for hsa-miR-17 and hsa-miR-107 are ranged from 0.65 to 0.8542 (**Figures 8B-D**) among different datasets. Based on our ceRNA network, the above results further suggested that these two regulator axes played key roles in type 1 diabetes, which may serve as potential therapeutic targets and diagnostic markers.

## Discussion

The prevalence and incidence of complex metabolic disease, such as type 1 diabetes and type 2 diabetes, is increasing worldwide. The pathophysiology and treatment may differ between them. Early diagnosis of diabetes by the usefulness of biomarkers can delay the onset of serious complications. In this study, based on the non-coding RNA and gene expression data of human islet exosome, we constructed networks combined with the regulatory associations of candidate lncRNA-miRNA and miRNA-mRNA, lncRNA-related ceRNA analysis networks.Our data revealed potential markers of type 1 diabetes, including lncRNA PVT1, LINC00960, hsa-miR-107 and elucidated the mechanisms they may involved in. At the same time, the proportion of different lncRNAs and mRNAs in the two networks was analyzed, and it was found that the proportion of different lncRNAs and mRNAs in the two networks was very small, and the proportion of different mRNAs was higher than that of different lncRNAs, suggesting that lncRNAs affected downstream mRNAs through differential regulation and thus affected functions. It can lead to the occurrence of diabetes and related complications. On the other hand, these analysis results also indicate that the lncRNA-related competitive regulatory network constructed in this study can provide certain supplements for the discovery of new diabetes diagnostic markers.

Considering the convenience of exosome and miRNA detection, it is a marker that can be widely promoted. To date, numerous studies revealed that exosomal miRNAs (such as miRNA-125b, miRNA-144, let-7, miRNA-155, miRNA-29, miRNA-133a, and miRNA-7) promote the development and progression of diabetes (28).Therefore, in this study, combined with miRNAs potentially mediating competitive regulation of pancreatic tissue exosomes in different states and their differential expressions, potential diagnostic markers of diabetes were explored and verified in different independent datasets of plasma or saliva exosome datasets. Among them, the miRNA markers found need to be further verified by experiments.

Finally, we analyzed the role of lncRNA in diabetes and related mechanisms based on the competitive regulation and downstream function of lncRNA-miRNA-mRNA-pathway. After performing bioinformatics analysis, the ceRNA regulatory network associated to pancreas-derived exosomes diagnosis of type 1 diabetes was obtained. It can provide a new reference and supplement for the study of diabetes-related mechanisms.

## Conclusion

This study is of great significance for the mechanism study of diabetes and its complications, the discovery of new diagnostic markers and therapeutic targets.

## Data availability statement

The original contributions presented in the study are included in the article/supplementary material. Further inquiries can be directed to the corresponding author.

## Ethics statement

Ethical review and approval was not required for the study on human participants in accordance with the local legislation and institutional requirements. Written informed consent for participation was not required for this study in accordance with the national legislation and the institutional requirements.

## Author contributions

TF, GX and WJ: Conceptualization, investigation, methodology, writing-review and editing. LW and ZX: Software, supervision, writing—original draft, writing—review and editing. YF: Conceptualization, resources, supervision, visualization, writing—original draft, Data curation, formal analysis, funding acquisition, investigation and writing-review and editing. All authors contributed to the article and approved the submitted version.

## Funding

This study was supported by National Natural Science Foundation (No.81901853) from YF. This study also was supported by Fund of Scientific Research Innovation of The First Affiliated Hospital of Harbin Medical University (2017B014) and Financial Assistance under Heilongjiang Postdoctoral Fund (LBH-Z18143) from TF.

## Conflict of interest

The authors declare that the research was conducted in the absence of any commercial or financial relationships that could be construed as a potential conflict of interest.

## Publisher’s note

All claims expressed in this article are solely those of the authors and do not necessarily represent those of their affiliated organizations, or those of the publisher, the editors and the reviewers. Any product that may be evaluated in this article, or claim that may be made by its manufacturer, is not guaranteed or endorsed by the publisher.

## Abbreviations

LncRNA, long-chain non-coding RNA; MiRNA, microRNAs; ncRNA, non-coding RNAs; GDM, gestational diabetes mellitus; GEO, Gene Expression Omnibus; Mst1,Mammalian sterile 20-like kinase 1; PVT1, plasmacytoma variant translocation gene; FOXO, Forkhead box class O; HIF, hypoxia-inducible factors; DPN, diabetic peripheral neuropathy; AUC, area under the curve.
